# Supplementary material for: Bacterial genome architecture shapes global transcriptional regulation by DNA supercoiling
Source: Nucleic Acids Res. 2019 Apr 24;47(11):5648–57. doi: 10.1093/nar/gkz300 (PMC6582348; doi:10.1093/nar/gkz300)
Supplement: gkz300_Supplemental_Files [file gkz300_supplemental_files.zip › sm.pdf]

Bacterial genome architecture shapes global transcriptional regulation by DNA supercoiling

Supplementary Material

Bilal El Houdaigui, Raphaël Forquet, Thomas Hindré, Dominique Schneider,
William Nasser, Sylvie Reverchon and Sam Meyer

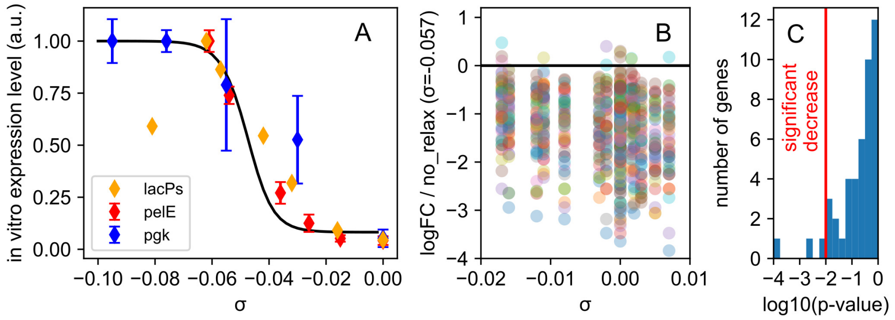

Figure 1: **(A)** The promoter activation curve used in the TSC model (Fig. 1C) reproduces the *in vitro* expression profile of bacterial promoters from several species (*pelE* from *Dickeya dadantii* [1], *lacPs* from *Escherichia coli* [2] and *pgk* from *Chlamydia trachomatis* [3]). The datapoints are superposed at $\sigma = 0$. **(B)** The *in vivo* expression profiles of the 46 most strongly relaxation-repressed genes in *Escherichia coli* [4] (different colours) confirm that the expression levels are very similar in the 15 different relaxed states (from $\sigma = -0.02$ to $\sigma = 0.01$) analysed in these data, whereas the repressive effect occurs between at supercoiling levels $\sigma < -0.02$ (the black line indicates the expression level before relaxation, $\sigma = -0.057$), as we assume in our model (see A). Note that these profiles are more difficult to interpret than *in vitro* assays, since the *local* SC level experienced by each chromosomal promoter differs from the *global* level measured on a plasmid, and transcriptomics experiment only give access to *relative* expression levels. **(C)** A statistical analysis of each independent promoter of B confirms that, in spite of a strong sensitivity compared to the non-relaxed SC level, the large majority exhibits no detectable expression variation for $\sigma \geq -0.017$ (only 3 show a significant decrease).

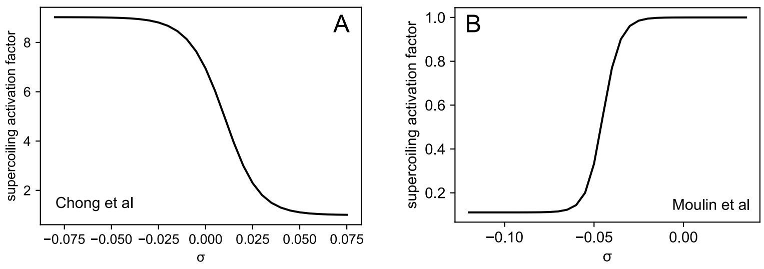

Figure 2: **(A)** Shifted effective promoter activation curve used to simulate the data of Chong et *al.* [5] (Fig. 2B). This modified curve accounts for the stalling effect of positive supercoils on transcription elongation in this *in vitro* experiment in absence of DNA gyrase, and is consistent with the observed repressive effect of positive supercoils. **(B)** Reversed promoter activation curve used for the *gyrA* promoter (Fig. 3B), which is a very specific promoter activated by DNA relaxation to ensure an homeostasis of the SC level in the cell [6].

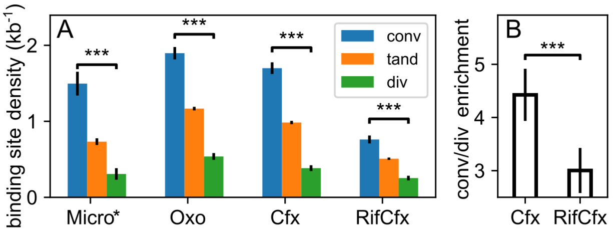

Figure 3: High-resolution chromosomal distribution of DNA gyrase activity sites in *Escherichia coli* [7] validates the contribution of TSC. **(A)** Activity sites mapped with different trapping agents (microcin, oxolinic acid, ciprofloxacin, ciprofloxacin+rifampicin) show a similar enrichment in convergent vs divergent regions of the genome. For microcin (*), the densities were multiplied by 10 for visibility. Intragenic binding sites were classified with respect to neighbour gene orientations. **(B)** Transcription inhibitor rifampicin significantly reduces the difference in activity between convergent and divergent regions (as computed from A), directly exhibiting a contribution of transcription in this effect, in addition to putative sequence-dependent differences in affinity. Note that, since the drug was applied for only 15 min, the RifCfx sample might still also contain a weaker contribution from SC induced by earlier transcription, not yet dissipated by topoisomerases.

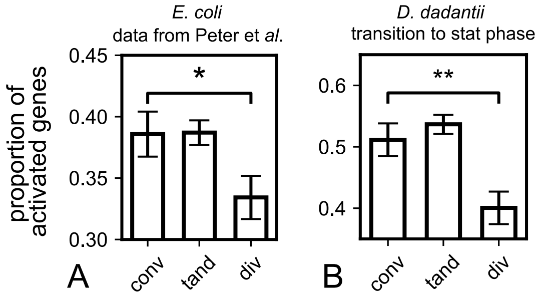

Figure 4: Additional analyses of genes' response to DNA relaxation. **(A)** Same as Fig. 4D, for the novobiocin data of Peter et *al.* [4]. For this experiment, the authors only provided global p-values obtained from many experimental conditions involving topological variations, resulting in a low number of significantly affected genes. For consistency with the other data presented here, we focused on the response to novobiocin treatment, for which the gene expression levels were provided but not the activation p-values. We therefore considered all genes as activated or repressed, explaining why the proportions are much closer to each other in this graph (vertical scale), but with narrower confidence intervals (larger number of genes in the dataset). **(B)** Effect of gyrase inhibition on *Dickeya dadantii* transcriptome at transition to stationary phase. Although the global expression pattern is very different from exponential phase (Fig. 4E), chromosomal relaxation exhibits the same selective activation of convergent vs divergent genes.

References

[1] Zghidi-Abouzid Ouafa, Sylvie Reverchon, Thomas Lautier, Georgi Muskhelishvili, and William Nasser (2012). The nucleoid-associated proteins H-NS and FIS modulate the DNA supercoiling response of the pel genes, the major virulence factors in the plant pathogen bacterium Dickeya dadantii. *Nucl Ac Res*, 40(10):4306–4319

[2] J. A. Borowiec and J. D. Gralla (1987). All three elements of the lac ps promoter mediate its transcriptional response to DNA supercoiling. *J Mol Biol*, 195(1):89–97

[3] Eike Niehus, Eric Cheng, and Ming Tan (2008). DNA Supercoiling-Dependent Gene Regulation in Chlamydia. *J Bact*, 190(19):6419–6427

[4] Brian J Peter, Javier Arsuaga, A M Breier, A B Khodursky, Patrick O Brown, and Nicholas R Cozzarelli (2004). Genomic transcriptional response to loss of chromosomal supercoiling in Escherichia coli. *Gen Biol*, 5(11):R87

[5] Shasha Chong, Chongyi Chen, Hao Ge, and X. Sunney Xie (2014). Mechanism of Transcriptional Bursting in Bacteria. *Cell*, 158(2):314 – 326.

[6] Laurent Moulin, A Rachid Rahmouni, and Frédéric Boccard (2005). Topological insulators inhibit diffusion of transcription-induced positive supercoils in the chromosome of Escherichia coli. *Mol Microbiol*, 55(2):601–610.

[7] Dmitry Sutormin, Natalia Rubanova, Maria Logacheva, Dmitry Ghilarov, and Konstantin Severinov (2018). Single-nucleotide-resolution mapping of DNA gyrase cleavage sites across the Escherichia coli genome. *Nucl Ac Res*.
